# Effects of Cisplatin in Neuroblastoma Rat Cells: Damage to Cellular Organelles

**DOI:** 10.1155/2012/424072

**Published:** 2012-02-28

**Authors:** Giada Santin, Luigi Scietti, Paola Veneroni, Sergio Barni, Graziella Bernocchi, Maria Grazia Bottone

**Affiliations:** ^1^Istituto di Genetica Molecolare, CNR, 27100 Pavia, Italy; ^2^Laboratorio di Biologia Cellulare e Neurobiologia, Dipartimento di Biologia Animale, Università di Pavia, Via Ferrata, 27100 Pavia, Italy; ^3^Laboratorio di Anatomia Comparata e Citologia, Dipartimento di Biologia Animale, Università di Pavia, 27100 Pavia, Italy

## Abstract

Cisplatin (cisPt) is a chemotherapy agent used as a treatment for several types of cancer. The main cytotoxic effect of cisplatin is generally accepted to be DNA damage. Recently, the mechanism by which cisPt generates the cascade of events involved in the apoptotic process has been demonstrated. In particular it has been shown that some organelles are cisPt target and are involved in cell death. This paper aims to describe the morphological and functional changes of the Golgi apparatus and lysosomes during apoptosis induced in neuronal rat cells (B50) by cisplatin. The results obtained show that the cellular organelles are the target of cisPt, so their damage can induce cell death.

## 1. Introduction

From many years, cisplatin (cisPt) was used in chemotherapy of different cancers because of its apoptotic effects. It binds DNA-generating adduct cisPt/DNA that provokes the distortion of double helix blocking the transcription and the replication processes [1]. We reported that, in B50 neuroblastoma rat cells, cisPt induces cytotoxic cell death mediated by activation of death receptor-mediated apoptotic signaling mechanisms as well as mitochondrial pathways [2, 3]. Nevertheless, organelles damage cisPt induced is today not so well elucidated. The cytotoxic action of this drug can be initiated by cytoplasmic events thus determining organelle damage. Yu et al. [4] demonstrated, in fact, that cisplatin initiates apoptosis from the cytoplasm and suggested that nuclear events may not be critical for the initiation of cisplatin-induced cytotoxicity, at least, not in immortalized mouse kidney proximal tubule epithelial (TKPTS) cells [4].

Apoptosis is a genetically controlled cell death program consisting of several essential steps that are critical checkpoints, as well as nonessential steps depending on the cell type, context, or pathophysiological stimuli [5]. Moreover, the intricate network of relationships and communication (i.e., crosstalk in which multiple organelles emit signals and receive responses) occurs between all the cellular organelles. During apoptosis, the cytoskeleton undergoes disassembly, bringing with him the different organelles, such as the Golgi vesicles, which in apoptosis following the reorganization of microtubules [6–8] wasting a clear spatial organization in the cell [9, 10]. Mitochondria and endoplasmic reticulum are, respectively, involved in intrinsic apoptotic pathway and in the pathway mediated by caspase 12 [11].

We have demonstrated that in B50 neuronal rat cells cisplatin induces apoptosis, morphological and functional modification of cytoskeleton, mitochondria [12], and endoplasmic reticulum [2], yet. In this work we applied histochemical techniques in confocal microscopy and electron microscopy to the analyses of changes of Golgi apparatus and lysosomes in B50 cells after treatment with cisPt.

## 2. Material and Methods

### 2.1. Cells and Treatments

B50 neuroblastoma rat cells (Istituto Zooprofilattico Sperimentale della Lombardia e dell'Emilia Romagna, cat. n. BS TCL 115) were cultured in “75 cm^2^” flasks in Dulbecco's minimal essential medium supplemented with “10%” fetal bovine serum, “1%” glutamine, “100 U” penicillin, and streptomycin (Celbio) in a “5% CO_2_” humidified atmosphere. “24 h” before experiments, cells were seeded on glass coverslips for fluorescence microscopy, or grown in “75 cm^2^” plastic flasks for electron microscopy analysis.

Cells are submitted to a continued exposure to cisPt (Teva Pharma) “40 *μ*M” for “48 h” at “37°C”. This concentration was chosen considering our in vivo experimental design (i.e., a single injection of “5 *μ*g/g” b. w. in “10-day-old” rats); this dose corresponds to the dose most commonly used in chemotherapy [13, 14]. For immunocytochemical analysis, after the treatments, the samples grown on coverslips were fixed with “4%” formalin and postfixed with “70%” ethanol for “30 min” each, at “−20°C.”

### 2.2. Double Immunocytochemical Detection of Golgi Proteins and Cytoskeletal Components

Cells were incubated with a solution of anti-Golgi proteins (autoimmune serum recognizing the Golgi proteins, a kind gift of the IRCCS San Matteo, Pavia, Italy) diluted “1 : 400” in PBS and another primary antibody: Alexa 594-Phalloidin (Molecular Probes, Invitrogen) diluted “1 : 40” in PBS. After “60 min” at room temperature, coverslips were incubated with the secondary antibody: Alexa 488-conjugated anti-human antibody (Molecular Probes, Invitrogen) diluted “1 : 200” in PBS for Golgi protein for “1 h.” Sections were counterstained for DNA with “0.1 *μ*g/mL” Hoechst “33258,” washed with PBS, and mounted in a drop of Mowiol (Calbiochem), for confocal microscopy analysis. Three independent experiments were carried out.

### 2.3. Double Immunocytochemical Detection of Golgi Proteins and Endoplasmic Reticulum

Cells were incubated with a solution of anti-Golgi proteins (autoimmune serum recognizing the Golgi proteins, a kind gift of the IRCCS San Matteo, Pavia, Italy) diluted “1 : 400” in PBS and another primary antibody: antireticulum proteins (Abcam) diluted “1 : 100” in PBS. After “60 min” at room temperature, coverslips were incubated with the secondary antibodies: Alexa 488-conjugated anti-human antibody (Molecular Probes, Invitrogen) diluted “1 : 200” in PBS for Golgi protein and Alexa-594-coniugated anti-rabbit antibody for reticulum proteins for “1 h.” Sections were counterstained for DNA with “0.1 *μ*g/mL” Hoechst “33258,” washed with PBS and mounted in a drop of Mowiol (Calbiochem), for confocal microscopy analysis. Three independent experiments were carried out.

### 2.4. Golgi Apparatus and Endoplasmic Reticulum Morphological Features at Transmission Electron Microscopy (TEM)

For TEM analysis, the cells grown in “75 cm^2^” plastic flasks for electron microscopy harvested by mild trypsinization (“0.25%” trypsin in PBS containing “0.05%” EDTA), immediately fixed with “2%” glutaraldehyde in the culture medium (“1 h” at “4°C”) and postfixed in “1% OsO_4_” in PBS for “1 h” at room temperature. The cell pellets were embedded in “2%” agar, thoroughly rinsed with Sörensen buffer (“pH 7.2”) and dehydrated in ethanol. Finally, the pellets were embedded in Epon resin and polymerized at “60°C” for “24 h.” Ultrathin sections were stained with uranyl acetate and lead citrate and observed under a Zeiss EM900 transmission electron microscope.

### 2.5. Double Immunocytochemical Detection of Lysosomes Proteins and Mitochondria

Cells were incubated with a solution of antilysosomes proteins (autoimmune serum recognizing the lysosome proteins, a kind gift of the IRCCS San Matteo, Pavia, Italy) diluted “1 : 500” in PBS and another primary antibody: mitochondrial anti-HSP70 (Molecular Probes, Invitrogen) diluted “1 : 50” in PBS. After “60 min” at room temperature, coverslips were incubated with the secondary antibody: Alexa 594-conjugated anti-human antibody (Molecular Probes, Invitrogen) diluted “1 : 200” in PBS for lysosome proteins and Alexa 488-coniugated anti-mouse for HSP70 for “1 h.” Sections were counterstained for DNA with “0.1 *μ*g/mL” Hoechst “33258”, washed with PBS, and mounted in a drop of Mowiol (Calbiochem), for confocal microscopy analysis. Three independent experiments were carried out. Confocal microscope software was used to obtain bar charts of colocalization; eight fields of cells were considered to count number of lysosomes per cells, both in control and in treated sample, and the graphic was done with excel program.

### 2.6. Confocal Fluorescence Microscopy

For confocal laser scanning microscopy, Leica TCS-SP system (Leica, Heidelberg, Germany) mounted on a Leica DMIRBE-inverted microscope was used. For fluorescence excitation, an Ar/UV laser at “364 nm” was used for Hoechst “33258”, an Ar/Vis laser at “488 nm” was used for FITC, and an He/Ne laser at “543 nm” was used for Alexa 594. Spaced (“0.5 *μ*m”) optical sections were recorded using a “63×” oil immersion objective. Images were collected in the “1024 × 1024 pixel” format, stored on a magnetic mass memory and processed by Leica confocal software.

## 3. Results

Our experiments revealed that cisPt treatment entails an organellar degradation and a reorganization in the cytoplasm. In particular, Golgi apparatus, endoplasmic reticulum, and lysosomes are the main target of the drug.

**Figure 1:**
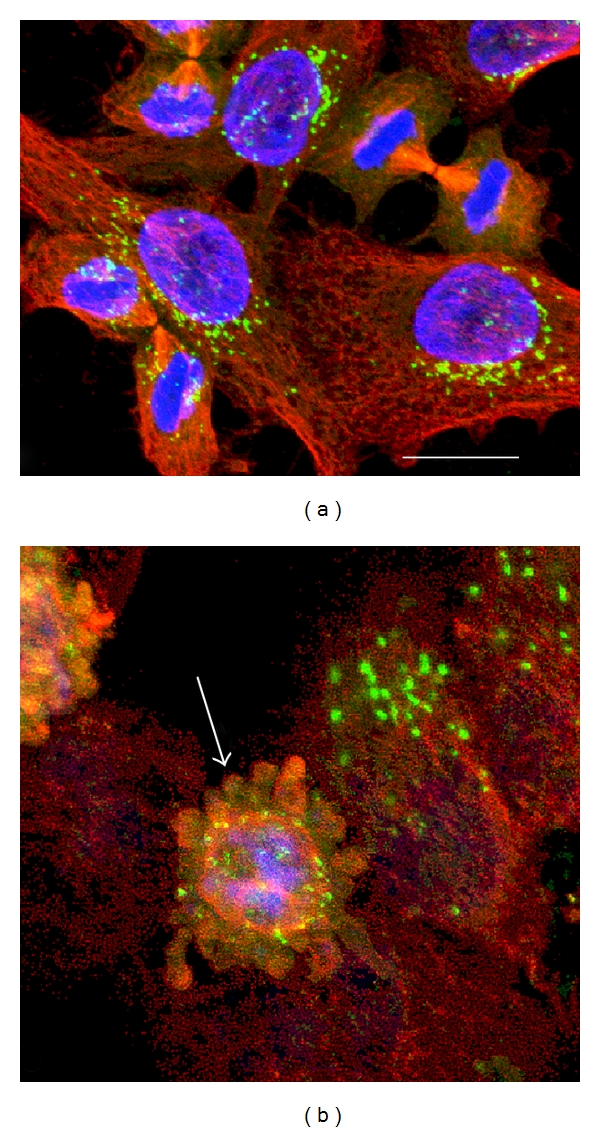
Double immunolabeling of Golgi apparatus (green fluorescence) and *α*-tubulin (red fluorescence) in B50 control cells (a) and in 48 h cisPt-treated cells (b). In (b), arrow indicates an apoptotic cell. DNA is counterstained with Hoechst 33258 (blue fluorescence). Bar: 20 *μ*m.

Figure 1 shows the Golgi apparatus (green fluorescence) in control cells (a) and in 48 h cisPt-treated cells (b); in control cells, Golgi exhibits its typical shape “ribbon like,” with flattened cisternae, according to its function as a proteins sorter. It mostly has a perinuclear localization in the cytoplasm with a progressive decreased concentration towards the periphery of the cell. In cisPt treated cells, a strong production of vesicles is visible, accompanied by a spatial redistribution and dense masses. After treatment, the tubulin cytoskeleton (red fluorescence) shows morphological changes, too. Soldani et al. [15] demonstrated that the morphological alterations of the Golgi apparatus may be related to structural changes in cytoskeletal apparatus [15]; in the treated cells, in fact, it can be observed that the vesiculation of this organelle is localized mainly at the periphery of the cell (arrow).

**Figure 2:**
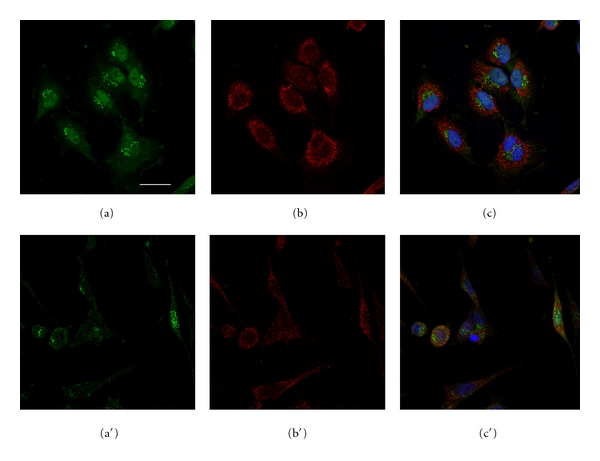
Double immunolabeling of Golgi apparatus (green fluorescence) and endoplasmic reticulum (red fluorescence) in B50 control cells (a, b, and c) and in 48 h cisPt-treated cells (a′, b′, and c′). In (c) and (c′), DNA is counterstained with Hoechst 33258 (blue fluorescence). Bar: 20 *μ*m.

 It is well known that the endoplasmic reticulum and Golgi apparatus are closely linked in terms of both organizational and functional point of view in the process of synthesis and posttranslational maturation of proteins through an intense vesicular traffic. In Figure 2, the double immunoexpression of Golgi apparatus and endoplasmic reticulum is shown both in control cells (a, b, and c) and in treated cells (a′, b′, and c′). In the first condition, Golgi apparatus maintains the perinuclear location (a) as it is above described in Figure 1, and endoplasmic reticulum is homogeneously arranged in the whole cytoplasm (b), where it is main concentrated near the nucleus. In the second condition, Golgi apparatus undergoes a strong disruption losing the typical semilunar shape (a′), while endoplasmic reticulum is less thickened and quite disrupted, too (b′).

**Figure 3:**
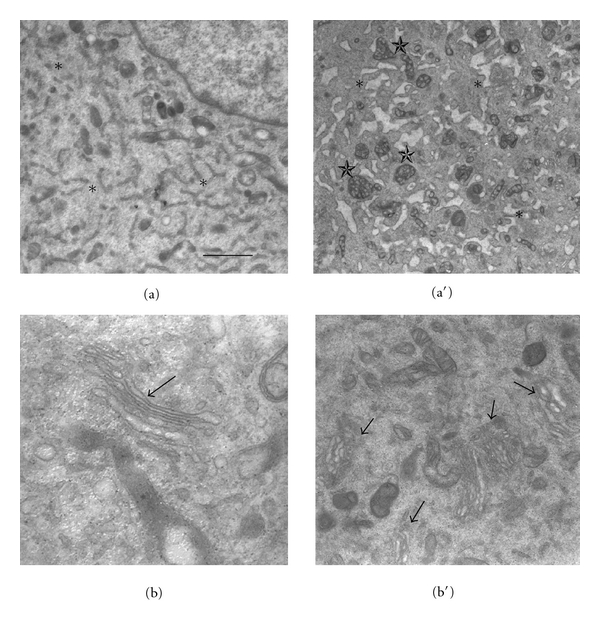
Cellular morphology at TEM in B50 control cells (a and b) and in 48 h cisPt-treated cells (a′ and b′). In (a and a′), asterisks indicatesendoplasmic reticulum; in (a′), stars indicate damaged mitochondria. In (b and b′), arrows indicate Golgi apparatus. Bar: 6 *μ*m.

Figure 3 depicts the ultrastructural morphology of B50 control (a and b) and treated (a′ and b′) cells at TEM. In (a) it is visible a cellular area with the intact endoplasmic reticulum (asterisks) properly distributed in the cytoplasm, but after treatment (a′) it results heavily dilated (asterisks). In (a′) it is also appreciable the mitochondrial damage (stars) cisPt induced [12]. In (b) Golgi apparatus in physiological condition is shown (arrow), but after treatment (b′) it results suffering, because it loses the regular shape of cisternae which becomes in some case dilated and in other more thickened (arrows).

**Figure 4:**
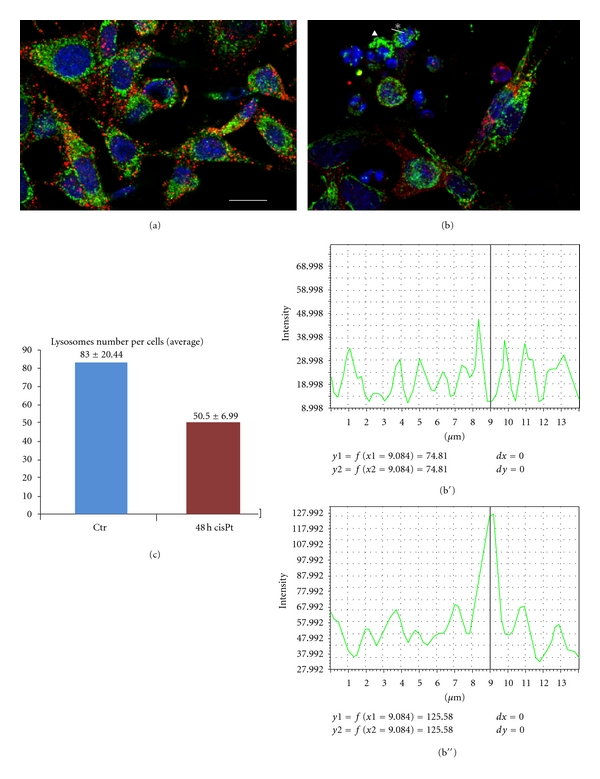
Double immunolabeling of lysosomes (red fluorescence) and mitochondria (green fluorescence) in B50 control cells (a) and in 48 h cisPt-treated cells (b). DNA is counterstained with Hoechst 33258 (blue fluorescence). Bar: 20 *μ*m. In (c), bar chart of lysosomes number per cells (average). In b, triangle represents dense mitochondrial masses, asterisk represents the selected point of noncolocalization. In (b′, b′′), bar charts of colocalization, the (b′) bar chart is for red fluorescence, the (b′′) is for green fluorescence.

Lysosomes are cisPt target, too. In Figure 4 the double immunolabeling of lysosomes (red fluorescence) and mitochondria (green fluorescence) is reported, compared to a control situation (a), where lysosomes are numerous, and mitochondria presents their typical cytoplasmic distribution, after cisPt injury (b) cells show a decrease of lysosomes number (c: from  83 ± 20.44  to 50.5 ± 6.99; a reduction of about 30%) and mitochondria form dense perinuclear masses (b: triangle). Moreover, it has to be noted that lysosomal and mitochondrial expressions do not colocalized in treated cells (see bar charts of colocalization b′ and b′′ for the selected point of noncolocalization asterisk in b).

## 4. Discussion

It is generally accepted that the damage induced upon binding of cisPt with the DNA may inhibit transcription and/or DNA replication mechanisms [16]. The process of cisplatin-induced apoptosis is complex and involves multiple pathways [2, 12], and in literature several studies demonstrate that apoptosis induced by cisPt can also be DNA injury independent [17]. Nevertheless, in cisPt-induced cell death mitochondrial are considered perhaps a more important target than nuclear DNA damage [18], due to hydrolyzed cisPt that generates a positively charged metabolite which preferentially accumulates within the negatively charged mitochondria. Thus, the sensitivity of cells to cisplatin appears to correlate with both the density of mitochondria [19] and the mitochondrial membrane potential [20]. We have already demonstrated that cisPt targets, in addition to DNA, included the cytoskeleton (actin and tubulin) and mitochondria [12]. Recent data have shown that cisPt is able to induce cell death in different cell lines and that this implies the involvement of cytoplasmic organelles [11]. In addition, Nakagomi et al. [21] have identified the fragmentation of the Golgi apparatus during apoptosis as a marker of some neurodegenerative diseases [21]. The results obtained have allowed us to demonstrate the onset of organelles alterations after cisPt treatment in B50 neuroblastoma rat cells. In particular, we focused on Golgi apparatus and endoplasmic reticulum injury, organelles that make up the digestive vacuole of the cell [7]. Both of them have been called “stress sensor” as it has been shown that proteins on their membranes are able to trigger the caspase cascade by activating caspase-12 [2, 6, 8]. Our obtained results, both in confocal microscopy and in TEM, indicate that these organelles undergo strong rearrangement due to the 48 h cisPt treatment and that these alterations are linked to the reorganization of cytoskeletal tubulin. This evidence suggests that the whole process is also on the base of the generation of apoptotic bodies, designed for the ultimate phagocytosis.

The effect of cisPt has been also evident in lysosomes that become smaller and less numerous in treated cells. Because lysosomes are involved also in autophagy mechanisms, we applied a double immunolabeling of lysosomes and mitochondria in order to analyze if there was a colocalization between the two respective fluorescence. The absence of this colocalization after 48 h treatment indicates that cisPt does not induce any autophagic process as alternative response to the treatment. So, we can conclude that immunocytochemical techniques and the TEM are useful to demonstrate that B50 neuroblastoma cells undergo apoptosis through organelles injury, and not only as a consequent of DNA damage.
